# Using a theory-driven creative process to design a peri-urban on-site sanitation quality improvement intervention

**DOI:** 10.1186/s12889-019-6898-7

**Published:** 2019-05-14

**Authors:** James B. Tidwell, Jenala Chipungu, Roma Chilengi, Val Curtis, Robert Aunger

**Affiliations:** 1 London School of Hygiene & Tropical Medicine, Keppel St, London, WC1E 7HT UK; 2 Center for Infectious Disease Research in Zambia, Plot 34620, Alick Nkhata Road, Lusaka, Zambia

**Keywords:** Behavior change intervention, Applied behavioral science, Theory, Behavior Centered Design, Peri-urban, Sanitation, Demand

## Abstract

**Background:**

Behavior change interventions have been developed by drawing from many different theories using design processes of varying specificity. We describe the development of a behavior change intervention to improve on-site peri-urban sanitation quality in Lusaka, Zambia using the Behavior Centered Design (BCD) framework to explain the results of the process applied to improving the quality of shared peri-urban sanitation and compare them to similar interventions.

**Methods:**

We used the BCD behavioral determinants model to synthesize the data from our literature review and formative research. Then, we partnered with creative professionals using a design process to develop a theory-driven on-site peri-urban sanitation intervention. Particular attention was paid to the implications of using BCD for intervention development on improving its effectiveness, increasing the contributions to knowledge for other behaviors and settings, and advancing the discipline of applied behavioral science.

**Results:**

Based on findings from a literature review and formative research, we designed an intervention to encourage landlords to improve their toilets by making them more accessible, desirable, hygienic, and sustainable. The intervention involved landlords meeting in facilitated groups every 2 weeks with individual follow-up after each meeting. The meetings presented surprising “hidden camera”-style videos to reveal tenants’ perspectives, used participatory activities to help landlords reevaluate the benefits they would derive from improving sanitation on their plots, and provided practical guidance and mechanisms to facilitate the performance of construction and cleaning behaviors.

**Conclusions:**

Using the BCD framework provided an easy-to-follow intervention design process. The resulting intervention is highly creative and multi-faceted, with each element having a theoretical role in an explicit theory of change. The development of this theory-driven intervention advances applied behavioral science by facilitating evaluation of each of the behavior change techniques and the overall delivery mechanism hypothesized to change the target behaviors. This informs the adaptation of these findings to improving on-site sanitation in other settings and the iterative development of the BCD model, which can be used to more effectively change other behaviors.

**Electronic supplementary material:**

The online version of this article (10.1186/s12889-019-6898-7) contains supplementary material, which is available to authorized users.

## Background

### The problem of peri-urban sanitation

Poor peri-urban sanitation is a large and growing public health problem, and the lack of strong evidence for how to improve it will make it difficult to meet the sustainable development goal (SDG) 6.2 of safely managed sanitation for all. About 4.5 billion people lack access to safely managed sanitation globally, and 29% of those live in urban areas [1]. The population of peri-urban areas, partially defined as urban areas lacking adequate sanitation, experiences worse health outcomes than rural or other urban areas [2]. It is estimated that the peri-urban population will more than double to about 2 billion by 2035 [3].

Despite global progress in reducing open defecation, the prevalence of shared sanitation is actually increasing in many regions, and is common in peri-urban areas [4]. While the discussion of whether high-quality shared sanitation can be considered adequate is ongoing, it is clear that the quality of much shared sanitation so poor that it is unlikely to meet any established quality standard [5].

Funding and programs to improve peri-urban sanitation have largely consisted of supply-side initiatives such as government- or donor-driven infrastructure investment [6], and there was little rigorous evidence generated about what works to increase demand for the improvement of on-site sanitation in peri-urban settings before the project began [7], though a few cleaning-focused trials have been conducted recently [8, 9]. Sanitation marketing programs are common, but generally seek to create demand while simultaneously improving the available supply [10]. This makes randomized trials of sanitation marketing programs infeasible, so that little is known of their impact, or the impact of demand and supply components separately that is not subject to many competing explanations including measurement challenges or seasonal or secular trends [7]. Though a variety of programs have seen success in rural settings that could apply in urban or peri-urban settings, few have been tested there, and the task of behavior change in these settings may more difficult [11, 12].

This paper documents the theory-driven design process of creating an intervention to be evaluated in the future to produce such evidence for Lusaka, Zambia, with the potential to inform programming in other settings. About 70% of the 2 million residents of Lusaka, Zambia live in peri-urban areas [13]. Residents live on plots, either in landlord or tenant households, and typically share a pit latrine located on the plot. A description of the results of this process clarify how an intervention to improve shared peri-urban sanitation quality facilitated the advancement of empirical knowledge about the target behaviors as well as applied behavioral science in general.

### Use of a theoretically-driven intervention design process

Using behavioral science theories to address public health problems such as poor on-site peri-urban sanitation quality is difficult due to the many theories potentially relevant to this under-studied behavior and a lack of clear methods for how to best select from among them and apply them [14]. This difficulty is made worse by both the long-standing proliferation of theories from within applied behavioral science (ABS) and the recent broadening of disciplines from which it draws. Within ABS, arguments for the best way to advance its theoretical foundations and methods have included an overall unifying synthesis [15], intentional, direct comparisons of empirical results obtained from divergent theories and methods [16], and allowing theories and methods to simply proliferate or fall out of favor naturally [17]. Complicating this debate are new contributions from disciplines that directly impact ABS, including the spread of behavioral economics and advances within neuroscience [18], which have varied definitions, evaluation mechanisms, and intended explanatory scope for theories. For example, economic theories are often narrower than general behavioral frameworks [19], while those of neuroscience bring a distinct natural science approach [20]. Thus, for ABS to advance as a discipline, an approach that is based on the most useful theories from across disciplines integrated into a system that can be applied in practice is needed.

The task of selecting and applying theories from the wide range of available options is generally done in three ways. First, intervention development sometimes begins with a review of empirical findings, followed by a search for theories relevant to the kinds of results identified (e.g., [21]). These “theory-*aware*” interventions may be developed with their own “theories of change,” but these usually have little resemblance to the pre-existing theories from which they draw in a piecemeal manner, so their analysis can contribute little to advancing ABS theory. Second, behavioral determinants theories are sometimes used to provide a priori assumptions about what might influence behavior. These may come from a particular discipline (e.g., social psychology for the Health Belief Model [22]) or may be consolidated from a range of disciplines into a theory for a particular type of behavior (e.g., water, sanitation, and hygiene in the IBM-WASH model [23]), but there is no prescribed process leading from these determinants to an intervention package. Using these determinant identification theories, “theory-*based*” intervention development can contribute to the advancement of behavior-specific knowledge. But, null results yield little guidance into whether the wrong determinant was targeted or the wrong delivery mechanism or content was chosen when there is no explicit process guiding the entire process (e.g., behavioral design [24]). Third, more systematic “theory-*driven*” approaches move beyond determinants to prescribing processes for selecting mechanisms of change (e.g., the RANAS model [25]). These may use processes that are more prescribed [25] or open-ended (such as Behavioral Design [26]), and may include different determinants, but the common thread is that every aspect of the program development is specified by the approach.

We argue that the best way to advance ABS is by developing theory-driven interventions. Such a process allows the adaptation of findings to different settings, provides sufficient explanatory breadth to allow the investigation of new behaviors, allows integration of more narrowly-focused, behavior-specific theories, and facilitates rigorous evaluation of all potential points of failure in a process evaluation. While theories focusing narrowly on psychological determinants may be helpful, the context-specific nature of behavior [27] makes theories that do not capture these factors less useful. Theories that describe only certain kinds of behaviors (e.g., habits) or only specific behaviors (e.g., exercise) may generate important insights, but without an integration into a broader framework, they provide little guidance on investigating behaviors outside their specific domain. Behavior Centered Design (BCD) [28] is a framework that takes such a theory-driven approach, which generates knowledge about the targeted behavior setting that can be adapted to novel behaviors and settings. The overview of this process presented below will demonstrate the scope of potential learning from this intervention and serve as an example of creating a theory-driven intervention.

### Behavior Centered Design overview

BCD’s generic theory of behavior change is based on the reinforcement learning paradigm [29, 30]. Any behavior change intervention must make a change in the physical, social, or biological environment that serves as a stimulus (surprise), which alters the brain or body of an individual (revaluation), which leads to the selection of the desired behavior (performance), which is presumably rewarded [29, 30]. Surprise most clearly describes when conscious attention is drawn to a new stimulus, though it is possible to alter behavior through environmental changes processed only subconsciously [31]. The stimulus must then cause revaluation of the target behavior, either by making existing motives more salient or adding new motives to a behavior. Finally, the individual must select the desired action, and if the value of the behavioral reward is sufficient, continued performance of the behavior will be encouraged. For behaviors that have not been previously performed, behavior can be motivated even by an expected reward based on observing others’ personal rewards [32] (e.g., in the study setting, perhaps seeing others who are pleased with sanitation improvements they have made), or the anticipated approval of others [33] (perhaps making a new sanitation improvement because one perceives that others will approve of it. However, reinforcement learning fails when rewards (or punishments) are inconsistent, delayed, rare, or not clearly linked to the behavior [34, 35].

**Fig. 1:**
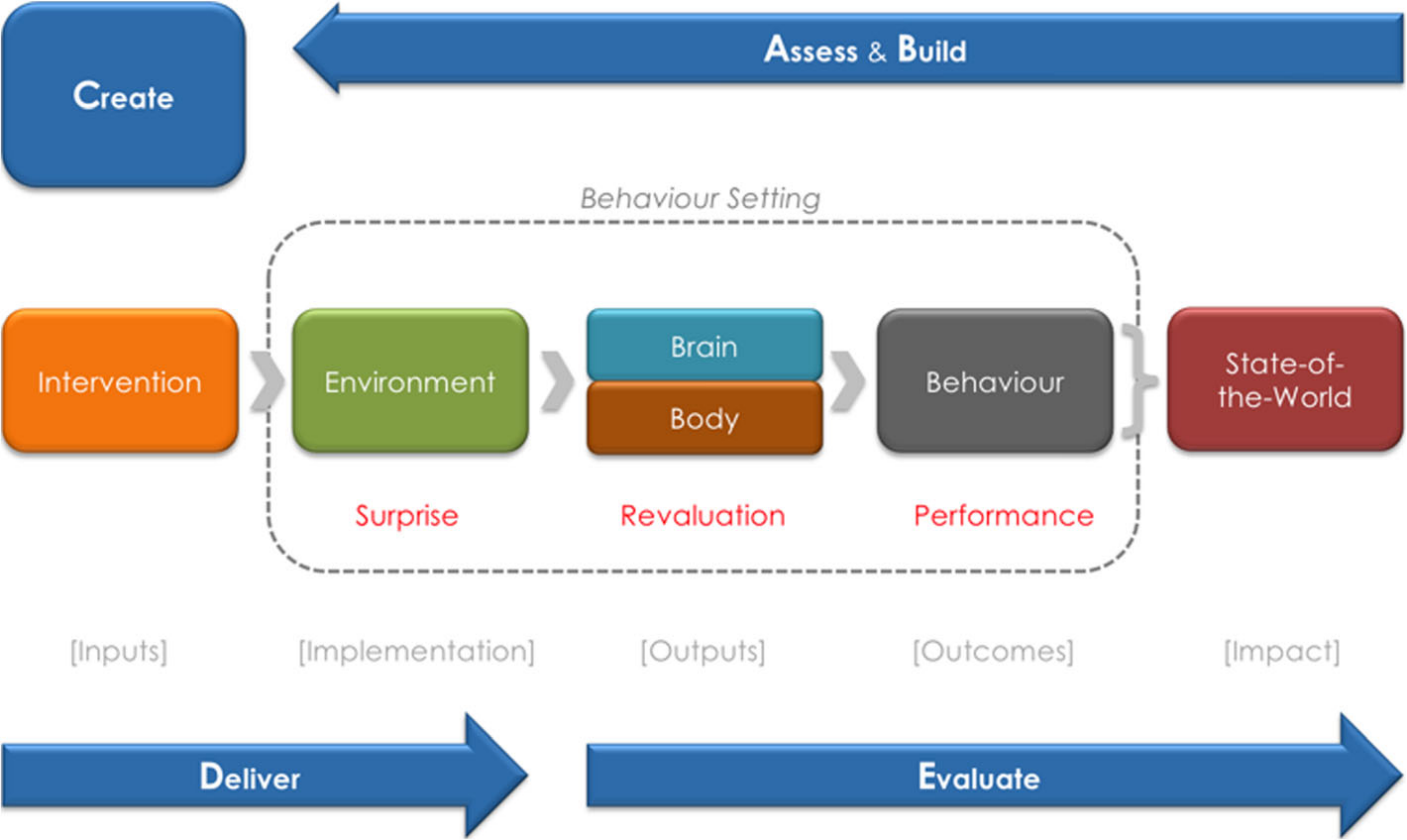
BCD Theory of Behavior Change and Design Process. The BCD design process works backwards from the desired change in the state of the world to find changes in behaviors, the brain/body, and the environment to be made through an intervention, which is then delivered and evaluated in reverse along the same theory of change. With Permission from Robert Aunger and Val Curtis from [28]

The BCD framework also uses a design process consisting of (1) Assessing existing knowledge, (2) Building knowledge to fill gaps identified, and then (3) Creating, (4) Delivering, and (5) Evaluating the intervention (Fig. 1). The overall process and findings from the first three steps of this “ABCDE” process for our example intervention are summarized in the results below.

## Methods

To *Assess* the state of knowledge about peri-urban sanitation intervention strategies, we conducted a systematized review of available literature on the drivers of on-site peri-urban sanitation improvement and included evidence from other settings as suggestive to supplement the limited research in peri-urban settings. We searched Medline, Embase, Scopus, Web of Science, and PubMed Central for the terms: (sanitation, toilet, or latrine) and (demand, motivation, driver, or determinant) and (slum, urban, or peri-urban). We limited the results to papers published prior to April 2016 (when the search was conducted), and also consulted local and international experts for grey literature pertinent to the topic. Lessons were drawn for an intervention designed to improve sanitation quality strictly through creating demand – that is, without relying on subsidy or provision of sanitation-related infrastructure.

For the Build step, we conducted formative research consisting of semi-structured interviews with landlords and tenants in the Bauleni neighborhood in Lusaka, Zambia (a peri-urban area). Respondents were asked about characteristics of their homes and shared areas on the plot with other residents, toilet construction histories, and the process by which investment and improvement of structures on the plot normally took place.

The Create step began with the study team hosting a creative workshop to present findings from the Assess and Build steps to representatives of 12 key stakeholder groups working in sanitation in Lusaka (see additional logistical details here: [36]). The initial day consisted of presenting the literature review and formative research findings along with extensive discussion.

During a brainstorm session on a second day, each attendee wrote down as many factors as they thought were important to understand how to create demand for sanitation on pieces of paper [37]. These factors were collected and laid out on the floor. As a group, those present sorted them into groups of similar factors; then participants came up with labels for these groups. This process was repeated to generate 15 high-level factor clusters, which were then placed by the attendees onto a two-dimensional set of axes representing causal importance and ease of change with respect to creating sanitation demand. From the high-ranking clusters a focal insight was then identified—“Your toilet is indecent (so you better do something to make it decent!)”—capturing the ideas that poor sanitation is disgusting and immoral; that tenants wanted better-constructed toilets, but landlords were unaware of this implicit demand, and that existing shared cleaning systems did not function well. Potential touchpoints (or contexts within which the target population might come into contact with the intervention) were also discussed.

The outputs of this process were compiled into a creative brief, which was presented to a local professional creative agency for the development of the campaign idea, content, and materials. This brief contained a broad array of findings from the formative research including key stakeholders, background, current situation, target audience, focal insights, theory of change, objectives, design principles, deliverables, budget, and timeline. Target improvements to latrine provision were selected for the intervention based on public health importance, association with diverse aspects of sanitation quality, the feasibility of making the improvement within the study timeframe, and the desire to include a variety of kinds of improvements with differing hypothesized determinants to test the effectiveness of different behavior change techniques through our delivery mechanism [38].

Several design principles were mandated by the project’s research objectives. First, the remit for this intervention was to determine the degree to which a behavior change approach could improve household sanitation quality with no action on the supply-side. Second, due to funding, the intervention period was to last only 6 months, so the targeted change had to be feasible within this period. Third, the outcomes had to be evaluated through an individually randomized design, and so mass media or whole-community approaches were excluded. This may have caused more effective approaches to be excluded, but the priority was to carefully evaluate a pilot that may later be scaled in other settings and that could be most reliably evaluated to understand the effects of this kind of intervention in isolation.

Additional design specifications coming from the formative research included targeting landlords rather than tenants, not focusing on health messaging, using a real-life tone in campaign materials, and framing the campaign with positive messaging. The intervention delivery mechanism design process was driven by the desire to engage the attention of the participants with a surprising message that caused revaluation of each behavior along with facilitating behavioral performance, corresponding to the Surprise, Revaluation, and Performance steps of the BCD theory of behavior change [28]. With reference to touchpoints, there was no place where landlords met exclusively for the purpose of interaction. Thus, places for mobilization had to be created.

The research and creative teams worked together to design the intervention through a series of revisions on the central intervention theme and delivery mechanism and the campaign manual, branding, and materials. Each stage was scrutinized based on the theoretical constructs of BCD to ensure that a streamlined intervention was produced whose evaluation would contribute to knowledge of peri-urban sanitation as well as improve the BCD process to advance ABS. The results of this creative process are described below.

## Results

### Assess and Build steps

Our literature review of peer-reviewed studies identified 6088 unique titles that were scanned, 317 of these whose abstracts were reviewed, and a final set of 60 full-texts were read and analyzed. Evidence for the impact of improving health knowledge was limited, with little evidence that it prompted adoption of better sanitation in Brazil [39] and its ranking below a variety of other factors for acquiring a higher-quality toilet in Senegal [40]. Comfort [41, 42], status [43, 44], fear [40, 45], disgust [46, 47], and affiliation [48, 49] were all suggested as motives for improving sanitation. Several studies focused on the importance of the social environment, whether through a sense of collective efficacy [9], direct peer influence [50, 51], or the role of community-level social networks [52]. Access to subsidies, financing, or existing financial wealth were also associated with better sanitation quality [53–56]. Land tenure security in peri-urban settings was found to be a strong determinant of sanitation quality [42, 57, 58].

Our formative research focused on the processes, roles, and priorities for landlords and tenants for improving peri-urban sanitation quality [59]. The key findings were:

- Landlords typically only made structural changes to toilets when existing structures got damaged or latrines got full or collapsed.
- Tenants were responsible for cleaning the toilet while landlords had the responsibility of financing the improvement of the physical elements of the toilet. However, if a tenant broke any features of a toilet (commonly door handle or lock) then they were responsible for replacing it.
- Landlords viewed their plots as a way to generate income, and would prioritize the building of another room to rent out than to improve their toilet.
- The top five toilet improvements identified were locking doors, sitting toilets, handwashing stands, lined pits, and smell reduction.
- With respects to shared sanitation roles, the relationships between landlords and tenants were weak, with landlords seeing tenants as the means to generate income from their plot and tenants feeling unable to express their desires to landlords.

Tenants expressed a willingness to pay (WTP) for these sanitation improvements through rental increases, but landlords underestimated this WTP and overestimated construction costs. Taboos surrounding the discussion of toilets likely contributed to landlords underestimating WTP. These gaps were quantified during the baseline data collection [60] and the results informed the intervention’s theory of change and were incorporated directly into the intervention messaging.

### Create step

#### Design process

The intervention design process combined the ABS expertise of the London School of Hygiene and Tropical Medicine (LSHTM) researchers, the local knowledge and experience of the team from the Centers for Infectious Disease Research in Zambia (CIDRZ), and the creativity of the local creative agency. The agency provided a series of five proposed versions of the campaign concept, two of printed materials, and two of live action videos for feedback; pre-tested campaign concepts and content with the target audience; and conducted a full pilot of the intervention with a single group of landlords. LSHTM and CIDRZ provided feedback on materials, pre-tests, and piloting based on research design considerations, formative research findings, and pre-test/piloting participant responses. They also streamlined the campaign manuals and lesson content based on the surprise/revaluation/performance paradigm and created emo- and exo-demos for behaviors that were lacking them.

The creative agency initially suggested several intervention ideas that had to be discarded as either logistically infeasible in the time allotted (creating a toilet evaluation system featured in a publication designed for tenants to browse homes for rent) or compromising the individually randomized evaluation plan (a “talking toilet” installed in the central market that drew attention to its own poor quality). Additional ideas, such as financial literacy training for landlords, were discarded when pre-testing revealed that most landlords viewed their plots as businesses, but this discovery led to a profit and conflict-reduction focused campaign focus.

Some concern was also raised among the team about profit-motive-related interventions and their potential to displace tenants through higher rental fees, resulting only in more income for landlords, or in the potential for increased marginalization caused by negative messaging [61]. We hypothesized that simply constructing high-quality toilets would inevitably lead to the displacement of many tenants. However, we established that many landlords did not think that tenants would pay for differences in sanitation quality [59] and that tenants were willing to pay [60], and thus focused on increasing the functioning of the market. There were few plots with low-quality housing and high-quality toilets, denying tenants the opportunity to access a plot with a higher-quality toilet, and while some tenants might not choose to or be able to pay more, the negative impact of allowing a larger range of choices was thought to be much smaller in this case. We also determined not to use any negatively-framed messages, instead focusing on the benefits that had been underestimated by landlords previously.

There was some tension between the expectations and processes of the creative agency and the researchers. These groups had very different perspectives on methodological rigor and expectations for the degree to which a campaign could change based on information from field testing. In addition, despite their “local” status, the intervention targeted areas of town and socio-economic classes a bit distant from some of the creative agency staff, and ensuring an adequate knowledge of and interaction with the target population was challenging. Though we prefer to involve local creative agencies, the BCD framework provided sufficient guidance to allow the researchers to generate additional intervention components and evaluate these components from both theoretical and practical perspectives, meaning that some components of the final intervention were produced by the creative agency, while others were generated by academic researchers.

The overall process from framing workshop to intervention delivery took 8 months, including 4 months to develop campaign concepts and materials and 4 months to complete video production. Several reasons for delays from the original 5-month timeline could be eliminated by other teams using this process to easily cut that timeline in half—we experienced slow administrative processes at both institutions and local government levels, procurement delays, and included an extended period for generating concepts based on the formative research data due to the lack of previously available information or interventions. Delivery to 20 groups of up to 25 landlords, with four meetings occurring over 2 months, was done by four pairs of presenters (one community health worker and one actor each), with tablets to show videos and some reusable printed materials. Four research assistants also worked as monitors. Materials provided to participants were only small printed cards and a durable plastic rota symbol, and rooms in local venues (churches and schools) were rented for delivery. Further details about study costs are available in the Additional file 1.

#### Intervention logic

The main intervention delivery mechanism was the creation of a “secret society,” which selected landlords would be invited to join so they could receive “insider knowledge” of how to build wealth and reduce conflict by improving sanitation on their plots. Meetings took place at a location near where they lived (either a school hall or church) rented for a small price by the project. Campaign manuals provided guidance for how to present “secret” information to landlords collected from tenants, some that allowed landlords to experience the emotions of their tenants, and some that gave practical tips. These were sorted by the researchers into the overall structure of surprise (videos), revaluation (games and demonstrations), and performance (practical guidance) for each meeting. These meetings were led by paid facilitators (often actors) trained in the intervention content as well as activation styles. High status was associated with meeting attendance and behavioral performance, as invitations were made using high-quality, branded materials and name badges displayed stars during the meetings to indicate the degree to which landlords made the targeted improvements.

The four target improvements identified were:

1. Regular cleaning of the toilet interface to reduce direct user exposure to pathogens
2. Installation of a lock on the inside of the door to increase safety and privacy
3. Installation of a lock on the outside of the door to allow access to plot residents while excluding outsiders
4. Installation of a water-sealed pan or cover to reduce smell and the spread of pathogens through vector contact with fecal material

The promoted locks, water sealed pans, and covers were already common in the local market. Regular latrine cleaning was encouraged by replacing a daily, verbal cleaning rotation with each household cleaning for a week, with a plastic decal hung above the door of the responsible household to bring accountability.

The opportunity for social reward, learning, and reinforcement was identified as a key behavior change mechanism, corresponding to surprise and revaluation in the BCD theory of change. Formative research indicated that landlords did not interact socially much with their tenants or even nearby landlords. In piloting, landlords praised even the opportunity to talk in an undirected manner about common challenges they faced as a helpful activity that rarely occurred otherwise. Hence, the intervention created a novel “social environment,” integrating aspects of learning by observing others from social learning theory [32]. In these meetings, landlords could learn from the successes of others, ask for advice from others in dealing with barriers faced, and in cases where few successes were reported in one group, stories from other group meetings could be used to provide additional insights. Landlords worked together to solve problems and to help each other to make improvements (the Affiliate motive), but were also given name badges with the stars indicating the quality of their toilet to bring a sense of hierarchy (Status) [62].

Another purpose of the four special-purpose group meetings was to facilitate behavioral performance through encouragement and monitoring. Paid monitors, distinct from the facilitators, conducted home visits to observe if improvements were made and to troubleshoot barriers faced. This information was given to the facilitators who used it for discussion at the start of the subsequent meeting. In addition, cards describing the main improvement were distributed to participants at the end of each meeting, which they were supposed to get a tenant to sign, indicating that they have taken relevant action after each meeting. These cards served as a tangible indicator of behavioral performance that could be monitored in the group setting, and visits by monitors to plots provided additional verification.

These cards also demonstrated the final purpose of the intervention structure—encouraging increased interaction between landlords and tenants to reveal unexpressed demand (again, surprise and revaluation). Each card represented a particular improvement that was made, and the required signature by a tenant designed to lead to an increasing number of discussions about working together in additional ways to improve sanitation on the plot. In particular, the card related to regular cleaning of the toilet (available at the project website [63]) required a signature verifying that a meeting had taken place between the landlord and his or her tenants for the explicit purpose of discussing a system for toilet cleaning.

Specific messages and activities were developed for each target behavior within the overall surprise, revaluation, and performance framework of each meeting’s content as well (Table 1). For “Surprise,” we chose to create live action, “hidden camera”-style videos, surprising participants with both edgy content that they may rarely observe (such as a man failing to aim properly while using a toilet due to his concern about holding a door closed) and information that is not generally communicated to them as landlords (such as tenants admitting that a poor toilet has scared them away from renting a room).

**Table 1:** Key Messages and Segment Content for Each Landlord Meeting

Outcome		Improved Cleaning Rota	Inside Lock	Outside Lock	Covered or Water-sealed Toilet
Surprise	Key Message	An improved rota keeps the toilet clean and makes your tenants happy.	Without an inside lock on your toilet, your tenants are robbed of their privacy.	A toilet without an outside lock will be abused by others and anger your tenants.	A smelly toilet full of flies will scare away paying tenants.
	Video Description	Tenants gossip about who doesn’t clean the toilet, and this boils over into full-blown conflict and blaming the landlord for not handling the problem.	Tenants struggle to keep the toilet door closed, culminating with a man walking in on a woman using the toilet. An argument ensues, and both end up blaming the landlord for the lack of a lock.	Drunk men stumble in to use the toilet at night, but when the landlord finds it dirty in the morning and yells at a tenant, she turns it back on him for not securing the toilet from outsiders.	A series of potential tenants come to look at a room for rent, ask to see the toilet, and then abruptly leave, confusing the landlord about what the problem was. The tenants privately discuss that they will go rent a more expensive place with a better toilet.
Revaluation	Key Message	Your toilet stays clean when the rota is simple and visible	A lack of privacy will drive good tenants away.	Asking tenants to do disgusting things will drive good tenants away.	A toilet is a wise investment that brings you more money quickly.
	Activity Description	Two teams were chosen with a landlord and 3 tenants each, and the tenants are assigned numbers—one team in blocks (i.e., 1–10) that are visible, and another in a more complicated, unwritten manner (i.e., every 3rd number). Landlords take turns identifying the tenant with a given number.	The facilitator asks for a chosen landlord to open their handbag and reveal every detail of the items inside and emphasizes the discomfort this lack of privacy causes.	Several participants were asked to come up one at a time to hold a tissue while the facilitator pretends to blow their nose loudly and messily. The facilitator translates this into the disgust tenants feel in having to clean up after outsiders who are messy and aren’t responsible to clean.	Two participants are assigned to invest either in improving the toilet or building a new room to rent. The toilet generates income sooner, rental gains are multiplied by the number of tenants, and a scenario where income is reduced shows that this is a more reliable and way to generate wealth.
Performance	Key Message	Give your tenants the power to remind one another of their responsibilities.	It is easy to install an inside lock by yourself or with your lock-buddy.	Remember to call on your ‘landlord lock-buddy’ to help you install an outside lock.	Invest in a decent cover pan (or a pour-flush toilet) to keep your plot full of tenants and build your wealth.
	Activity Description	Landlords are given a badge to hang outside the door of the tenant responsible for cleaning the toilet that week and asked to have a meeting with all tenants to institute the new system.	A handy man demonstrates installing a lock and then has landlords take turns practicing. “Lock buddies” are paired up to purchase and install locks.	Same as performance for inside lock.	A handyman describes how to build a simple cover and the process and cost of installing various flushing toilet options. Merry-go-rounds suggested to spread out the cost over time and encourage accountability to each other.

For revaluation, the SanDem intervention used “emo-demos,” or emotional demonstrations designed to revalue behaviors through emotional responses [64]. It also used “exo-demos,” an extension developed for this intervention, of “executive-level [i.e., cognitive] demonstrations” aimed at revaluing behaviors through activities requiring conscious group deliberation in areas such as calculating potential profits from toilet investments [65]. The overall theme of the revaluation sections was that a poor quality toilet leads a landlord to lose good tenants and give up a steadier, higher monthly rental income. Specific revealed desires of the tenants (e.g., privacy, cleanliness) were always translated directly into motivations for landlords (e.g., reduced plot conflict, more rental income). Exact details of the intervention can be found in the facilitator guide [63].

Behavior-specific performance facilitation varied by the kind of behavior. For cheaper, one-time actions (installing outside and inside locks), a buddy system was used where pairs of landlords helped each other purchase and install the locks at a set time following the meeting. For cheaper, ongoing actions (initiating an improved cleaning rota), an initial meeting with tenants was reported, and use of a visible, durable symbol of the cleaning system was verified during monitoring visits described below. For more expensive, one-time actions (improving the seal of the toilet or building a door, perhaps necessary prior to lock installation), a handyman already working in the community provided information about products available and the range of installation costs based on existing infrastructure. “Merry go rounds,” a common local mechanism where each participant contributed money each round and one participant received the contributions (rotating each round), were also suggested to landlords to ease the amount of one-time savings required and to provide peer accountability for making pledged improvements.

## Discussion

Using a theory-driven intervention development process improved the development of this intervention in two major ways. First, compilation of a collection of behavioral determinants, drawn from the empirical literature in the Assess step and from the BCD behavioral determinants model during the Build step, resulted in an in-depth understanding of the behaviors involved in sanitation improvement despite little previous work in the area. Hypotheses were generated for the local context unobserved in published literature on sanitation demand.

Second, the insight generation process produced a variety of creative ideas via a straightforward, but not deterministic, process. (Recent findings from evolutionary psychology applied to human reasoning suggest that generating possibilities, followed by deliberative reasoning to analyze them, as characteristic of BCD, is how the mind most naturally comes up with creative, workable ideas [65, 66].) Few program development processes provide guidance on how to generate appropriate insights or how to move from insights to intervention. BCD, however, borrows processes and tools from design thinking [67], including field testing of prototype interventions, to highlight specific contextual factors that may be important and suggests an iterative process for generating intervention ideas, analyzing them with specific practical and theoretical considerations, and repeating the process until an acceptable intervention that meets all the criteria of the creative brief is created. This process allowed creative agencies to do what they do best—come up with many novel, locally acceptable ideas—while allowing academics to do what they do best— ensure the intervention reflects principles from behavioral science and evaluation design for each piece of the intervention. Additional details about how to implement the process are available on the BCD website [68].

Reflection on the intervention development process has produced learning about the BCD design process itself—specifically, on how best to utilize creative agencies. At the beginning of the collaboration, the creative agency tended to move forward in ways that deviated from the formative research findings (such as health messages creeping into video dialogue), inserted typical campaign components not found in the brief (such as standard financial literacy training), drifted towards the flashy rather than the practical (such as an app rather than videos), or ignored research-specific requirements (such as only using plot-level components). When closely supervised and given specific guidance (such as developing a rota symbol with explicit design criteria), the agency excelled, and they were also amenable to feedback. The efficiency of the design process could be improved by increasing the degree of collaboration – e.g., by involving a creative agency team member in all aspects of formative research, requiring quicker reverts on smaller sections of the intervention, encouraging informal feedback from the research team after creative agency brainstorming sessions, and including a research team member in all material production meetings or video production activities. In this case, the whole process was facilitated by the research team having already had extensive experience with a variety of creative agencies.

It is not possible to ascertain whether this creative process produces a maximally effective intervention because creative processes can’t be compared based on program outcomes without incorporating multiple interventions, each produced using a different creative process, and delivered in the same way to the same population at the same time. Nevertheless, there are obvious strengths of the BCD process: it is based on that used by creative professionals (i.e., a design process); typically involves creative professionals (e.g., commercial creative firms) who are likely to be better at producing effective interventions than public health researchers or NGO members; is explicitly tied to a theory of change at all times (anchoring design processes to behavioural theory); and that theory allows for the broadest possible range of creative techniques and methods (including modification of environmental determinants -- e.g., product design, or physical ‘nudges’ such as our improved cleaning rota system).

For example, it is instructive to compare similar trials for their processes of designing peri-urban sanitation behavior change interventions. Two recent trials focussed on the cleanliness of shared on-site sanitation: One in Uganda, based on the RANAS model [25], where landlords and tenants participated in group discussions about toilet maintenance [9], and one in Bangladesh, based on IBM-WASH [23] and the Health Belief Model [22], where behavior change communications were used along with provision of cleaning materials to encourage cleaning behavior [8]. As noted, we are unable to make direct comparisons of empirical results, since any such comparisons would inevitably offer only limited information for reflecting on the relative merits of the approaches themselves, especially due to the drastically different contexts and outcome measures used. Instead, we reflect on two strengths of BCD compared to weakness observed in these other approaches. First, since BCD is a theory-*driven* approach, any failure to produce behavior change in our program requires that there were flaws with either the list of determinants, the process of identifying relevant determinants, or the process of moving from these to the intervention. A similar failure in the program in Bangladesh, using the theory-*based* IBM-WASH and Health Belief Model, could have little to do with the validity of the underlying theories, but instead could be a result of any number of failures to apply these theories appropriately (due to their lack of a program development process) or due to the lack of a theory of change (rather than simply a list of behavioral determinants) provided by either approach. Second, while the RANAS model used to develop the intervention in Uganda includes a wide range of behavioral determinants and a systematic process, it is designed to produce behavior change communication with messages tailored to the identified determinants, rather than taking advantage of BCD’s intentionally broad creative approach. Exploring a wider range of behavioral determinants and delivery mechanisms is likely over the long run to produce more effective interventions given the importance of choosing the right messengers and channels [69] to intervene on behaviors with complex, multi-level determinants [70].

## Conclusion

We used a theory-driven process based on the Behaviour Centred Design (BCD) framework to design an intervention to improve peri-urban sanitation quality. We followed the BCD steps of literature review (Assess), formative research (Build), and designing the intervention alongside creative professionals (Create), which resulted in an intervention that has explicit theories of change for each behavior and for the overall delivery mechanism. The intervention consisted of videos to reveal surprising information to landlords, repeated group meetings to create opportunities for social learning and revaluation of the target behaviors, and accountability mechanisms and a new cleaning system to facilitate behavioral performance.

This intervention was developed for a relatively little-studied behavior (demand-driven improvement of shared sanitation facilities) by adapting findings from similar kinds of behaviors as well as utilizing a generic framework for understanding human behavior. This theory-driven process produced an intervention that facilitates the advancement of applied behavioral science theory as well as knowledge related to this specific behavior.

## Additional file

Additional file 1: Program Costs. Additional detail about program-related costs are provided here to aid other implementers in understanding the costs of the approach followed. However, this program was designed as a pilot, and thus full cost analysis and formal cost-benefit analysis were not conducted at this stage, though attempts to take this program to scale (directly or through technologically enhanced means) should certainly collect this information. (DOCX 15 kb)

## Abbreviations

ABS: Applied Behavioral Science
BCD: Behavior Centered Design
CIDRZ: Center for Infectious Disease Research in Zambia
LSHTM: London School of Hygiene and Tropical Medicine
WTP: Willingness to Pay
