# Supplementary material for: Using a theory-driven creative process to design a peri-urban on-site sanitation quality improvement intervention
Source: BMC Public Health. 2019 May 14;19:565. doi: 10.1186/s12889-019-6898-7 (PMC6518808; doi:10.1186/s12889-019-6898-7)
Supplement: Supplementary file 1 — Program Costs. Additional detail about program-related costs are provided here to aid other implementers in understanding the costs of the approach followed. However, this program was designed as a pilot, and thus full cost analysis and formal cost-benefit analysis were not conducted at this stage, though attempts to take this program to scale (directly or through technologically enhanced means) should certainly collect this information. (DOCX 15 kb) [file 12889_2019_6898_MOESM1_ESM.docx]

# Supplementary Material

## Appendix A: Program Costs

Additional detail about program-related costs are provided here to aid other implementers in understanding the costs of the approach followed. However, this program was designed as a pilot, and thus full cost analysis and formal cost-benefit analysis were not conducted at this stage, though attempts to take this program to scale (directly or through technologically enhanced means) should certainly collect this information:

Table A1: Program Development and Delivery Costs

| **CREATIVE AGENCY** | **Unit Cost** |  |  |
| --- | --- | --- | --- |
| Creation and Filming | £26,000 | 1 | £26,000 |
| Materials Production | £12,000 | 1 | £12,000 |
| ***Subtotal*** | | | **£38,000** |
| **ACTIVATION** | **Unit Cost** |  |  |
| Presenters (8 for 4 months) | £225 | 32 | £7,200 |
| Monitors (4 for 3 months) | £185 | 16 | £2,960 |
| Activation Consultant | £1,000 | 9 | £9,000 |
| Rental costs (per venue) | £10 | 60 | £600 |
| ***Subtotal*** | | | **£19,760** |
| ***Total*** | | | **£57,760** |
